# PGM1 suppresses colorectal cancer cell migration and invasion by regulating the PI3K/AKT pathway

**DOI:** 10.1186/s12935-022-02545-7

**Published:** 2022-05-25

**Authors:** Zhewen Zheng, Xue Zhang, Jian Bai, Long Long, Di Liu, Yunfeng Zhou

**Affiliations:** 1 Department of Radiation Oncology and Medical Oncology, Zhongnan Hospital of Wuhan University, 169 Donghu Road, Wuchang District, Wuhan, Hubei People’s Republic of China; 2 Department of General Practice, Beijing Friendship Hospital, Capital Medical University, Beijing, 100050 People’s Republic of China; 3 Department of Anesthesiology, Peking University Third Hospital, Beijing, 100191 People’s Republic of China

**Keywords:** Phosphoglucomutase 1, Colorectal cancer, Prognosis, Proliferation, Apoptosis

## Abstract

**Background:**

Phosphoglucomutase 1 (PGM1) is known for its involvement in cancer pathogenesis. However, its biological role in colorectal cancer (CRC) has remained unknown. Here, we studied the functions and mechanisms of PGM1 in CRC.

**Methods:**

We verified *PGM-1* as a differentially expressed gene (DEG) by employing a comprehensive strategy of TCGA-COAD dataset mining and computational biology. Relative levels of PGM-1 in CRC tumors and adjoining peritumoral tissues were determined by qRT-PCR, western blotting (WB), and immunohistochemical (IHC) staining in a tissue microarray. PGM1 functions were analyzed by CCK8, EdU, colony formation, cell cycle, apoptosis, and Transwell migration and invasion assays. The influence of PGM1 was further investigated by studying tumor formation in vivo.

**Results:**

The levels of PGM1 mRNA and protein were both reduced in CRC tissues, and the reductions were related to CRC pathology and overall survival. PGM1 knockdown stimulated both cell proliferation and colony formation, and inhibited cell cycle arrest and apoptosis, while overexpression of PGM1 produced the opposite effects in CRC cells both in vivo and in vitro. Furthermore, the effects of PGM1 were related to the PI3K/ AKT pathway.

**Conclusion:**

We verified that PGM1 suppresses CRC progression via the PI3K/AKT pathway. These results suggest the potential for targeting PGM1 in treatment of CRC.

**Supplementary Information:**

The online version contains supplementary material available at 10.1186/s12935-022-02545-7.

## Background

Colorectal cancer (CRC) results in a high number of cancer-related deaths throughout the world [1]. In the USA, 147,950 new cases of CRC were reported in 2020 and approximately 53,200 deaths are reported annually [2]. The primary therapy options mainly depend on CRC staging. The five-year survival rate for early-stage disease exceeds 90% [1, 3]. Unfortunately, many patients already have advanced-stage disease at the time of diagnosis [4]. Therefore, there remains a need to identify reliable biomarkers and cancer-related molecular mechanisms that can assist with making decisions regarding a patient’s prognosis and developing suitable therapeutic strategies.

The microenvironment of a tumor plays a critical role in its metastatic spread. Increased glycolytic activity is associated with higher microenvironmental energy demands [5, 6] and tumors are characterized by a switch from oxidative phosphorylation to glycolysis as their main energy source [7]. Biosynthesis occurring within the cell supplies the necessary components and imparts a selective advantage to neoplastic cells [8]. Phosphoglucomutase (PGM) plays a key role in glucose metabolism [9], and the enzyme has been linked to cancer growth, metastasis, and invasion [10, 11]. Phosphoglucomutase 1 (PGM1) is encoded by the *PGM1* gene; PGM1 deficiency is a recognized inherited metabolic disorder (CDG1T) that is linked to a variety of diseases and disorders, including liver disease, exercise intolerance, and dilated cardiomyopathy, reflecting the key role played by the enzyme in glucose metabolism [12–14]. PGM1 inhibits hepatocellular carcinoma cell (HCC) proliferation and growth by utilizing sufficient extracellular glucose to convert glycogen, while deletion of the *PGM1* gene inhibits glycogen synthesis and leads to glycolysis of additional glucose, thus promoting tumor cell proliferation and growth [15]. However, PGM1 showed the opposite effect in lung cancer, under glucose depletion conditions, where researchers found that an upregulation of PGM1 increased glycogen content, and thereby reduced the rates of glycogen decomposition and glycogenesis, glycogen accumulation induces cell survival and proliferation under conditions of glucose depletion, decrease in PGM1 reduces tumor cell proliferation under prolonged glucose depletion [16]. Thus, PGM1 can play a tumor-promoting or anti-tumor-promoting role in an environmentally-dependent manner.

Here, we investigated the role of PGM1 and its regulation in CRC. We identified a new mechanism by which PGM1 suppresses CRC progression by regulating glucose translocation via the PI3K/AKT pathway.

## Materials and methods

### TCGA data sets

Differentially expressed genes were investigated using TCGA COAD (colorectal adenocarcinoma) and READ (rectal adenocarcinoma) data sets. The Ballgown R package in Bioconductor was used to analyze differential expression in RNA-seq data. Gene expression was compared between cancer and adjoining non-cancerous tissues. P-values and differences between the q-value and fold-change were calculated using the following criteria for differential expression: P < 0.01, q < 0.05, and fold- change > 2. GEPIA (gepia.cancer-pku.cn) was used for normalization and log2-scaling.

### Collection of clinical samples

The study protocol was approved by the Institutional Review Board of Wuhan University Zhongnan Hospital. Written informed consent was obtained from all patients prior to sample collection.

Samples (tumor and adjoining normal tissue) were collected from 76 patients with primary CRC who were admitted to Wuhan University Zhongnan Hospital between July 2019 and October 2020. The samples were kept frozen at − 80 °C until use. Patient clinical information is provided in Table 1.

**Table 1:** Associations of PGM1 expression with clinicopathological factors in CRC patients

Variable	Number	PGM1 expression		P-value
		High	Low	
		(N = 38)	(N = 38)	
Age(year)				
< 65	59 (77.6%)	31 (81.6%)	28 (73.7%)	0.582
≥ 65	17 (22.4%)	7 (18.4%)	10 (26.3%)	
Gender				
Female	32 (42.1%)	12 (31.6%)	20 (52.6%)	0.104
Male	44 (57.9%)	26 (68.4%)	18 (47.4%)	
Tumor size (cm)				
< 5	41 (53.9%)	26 (68.4%)	15 (39.5%)	0.021
≥ 5	35 (46.1%)	12 (31.6%)	23 (60.5%)	
Differentiation				
Moderate	19 (25.0%)	9 (23.7%)	10 (26.3%)	0.447
Poor	34 (44.7%)	15 (39.5%)	19 (50.0%)	
Well	23 (30.3%)	14 (36.8%)	9 (23.7%)	
Lymphatic node metastasis				
Negative	42 (55.3%)	28 (73.7%)	14 (36.8%)	0.003
Positive	34 (44.7%)	10 (26.3%)	24 (63.2%)	
TNM stage				
I + II	34 (44.7%)	25 (65.8%)	9 (23.7%)	< 0.001
III + IV	42 (55.3%)	13 (34.2%)	29 (76.3%)	
Distant metastasis				
No	44 (57.9%)	27 (71.1%)	17 (44.7%)	0.037
Yes	32 (42.1%)	11 (28.9%)	21 (55.3%)	
1 years of survival				0.003
Live	62 (81.6%)	36 (94.7%)	26 (68.4%)	
Dead	14 (18.4%)	2 (5.3%)	12 (31.6%)	
5 years of survival				< 0.001
Live	29 (38.2%)	23 (60.5%)	6 (15.8%)	
Dead	47 (61.8%)	15 (39.5%)	32 (84.2%)	

### Immunohistochemistry and scoring

Immunohistochemical analysis (IHC) was performed using a CRC tissue microarray (TMA) slide (Cat: HColA180Su10; Shanghai Outdo Biotech Co., Ltd., China). The TMA included CRC tissue specimens obtained from 100 surgically resected patients during the time period of April to November 2008. The patients were followed up until July 2015 (range, 6.7–7.2 years), had a mean age of 66.83 years (range 45–91 years), and included 58 men and 41 women. Table 1 shows the clinical data for these patients. Tissue sections (4 μm thick) were cut and deparaffinized. The sections were then microwaved for 5 min in citric acid (pH 6.0), incubated with an anti-PGM1 antibody (#15161-1-AP; Proteintech, China; 1:200), overnight at 4 °C, and then incubated with a secondary antibody. Images of the tissues were examined by two blinded pathologists and scored by multiplying the staining intensity (grades 0, 1, and 2 indicated negative; grade 3, weakly positive; grade 4, moderately positive; grade 5, strongly positive) by the positive rate score (score 0 = 0%, 1 = 0–5%, 2 = 6–25%, 3 = 26–50%, 4 = 51–75%, and 5 = 76–100%).

### Cell culture

Human CRC lines HT-29, LoVo, COLO205, SW620, and HCT116, together with the normal human colorectal cell line HCoEpic, were provided by the Institute of Biochemistry and Cell Biology of the Chinese Academy of Sciences (Shanghai, China). The cells were cultured in DMEM (Gibco, Waltham, MA, USA) supplemented with 10% fetal bovine serum (FBS; Gibco) at 37 °C in a humidified chamber containing 5% CO_2_.

### Western blotting

Western blotting was utilized to analyze PGM1 expression in tumor tissues and cultured cells. The cells and tissues were lysed in RIPA buffer (Beyotime, Beijing, China) containing proteolytic inhibitors (Genebase, Shanghai, China), and protein concentrations were measured with a Pierce BCA Protein Assay kit (Thermo Scientific, Waltham, MA, USA). Protein extracts (30 μg) were separated on 10–12% SDS-PAGE gels, and the separated protein bands were transferred onto polyvinylidene fluoride (PVDF) membranes (Millipore, Bedford, MA, USA). The blots were probed with primary antibodies including PGM1 (Abcam, Cambridge, UK; #232959; 1:1000 dilution), PI3K (Abcam; #ab32089; 1:10,000), p-PI3K (Abcam; #ab278545; 1:10,000), AKT (Abcam; #ab8805; 1:10,000), p-AKT (Abcam; #ab38449; 1:10,000), Bcl-2 (Abcam; #ab182858; 1:10,000), Bax (Abcam; #ab32503; 1:10,000), p21 (Abcam; #ab109199; 1:10,000), Cyclin D1 (Abcam; #ab40754; 1:10,000), and GAPDH (CST, Danvers, MA, USA; #3686; 1:1000) overnight at 4℃. Next, secondary antibodies were added at room temperature, 1 h), and immunostaining was detected with an ECL Western blotting Detection System (Amersham, Piscataway, NJ, USA). The loading control was GAPDH, and each blot was analyzed in triplicate. ImageJ software was used for a quantitative analysis of staining.

### RT-PCR

The total RNA was extracted cells and tissues using TRIzol Reagent (Invitrogen, Waltham, MA, USA), and cDNA was obtained using a Bestar™ qPCR RT kit (DBI Bioscience, #2220, Germany). The PGM1 primers were 5′-AGCATTCCGTATTTCCAGCAG-3′ (forward) and 5′-GCCAGTTGGGGTCTCA TACAAA-3′ (reverse). The GAPDH primers were 5′-TGTTCGTCATGGGTGTGAAC-3′ (forward) and 5′-ATGGCATGGACTGTGGT CAT-3′ (reverse). PGM1 expression was measured by the RT-PCR that was performed using Bestar™ qPCR MasterMix (DBI Bioscience, #2043, Germany). The control gene was *GAPDH*, and relative levels of gene expression were calculated by the 2^−ΔΔCt^ method [17].

### Immunofluorescence (IF)

Immunofluorescence staining of cells was performed as previously described [18]. In brief, cells were fixed in 4% paraformaldehyde for 15 min and then permeabilized with 0.1% Triton X-100 for 10 min at 4 °C. Next, the cells were incubated overnight with primary antibodies, followed by incubation with secondary antibodies (60 min, room temperature). DAPI was used for staining nuclei (5 min), and the sealed coverslips were evaluated with a laser scanning confocal microscope (Zeiss, Germany).

### Immunohistochemistry (IHC), Ki 67, and H&E staining

For IHC, tissue sections were deparaffinized and endogenous peroxide was inactivated. The sections were then blocked and incubated with anti-PGM1 (Proteintech; #15161-1-AP; 1:200) or anti-Ki67 (Bbcam; #ab15580; 1:400) primary antibodies and secondary antibodies (Abcam; #ab205718; 1:4000). For H&E staining, sections of mouse xenograft tumors were deparaffinized and then rehydrated and stained with H&E (Sigma-Aldrich, St. Louis, MO, USA) prior to dehydration and sealing. The sections were assessed and photographed under a phase contrast microscope (Leica, Cat. #DMI 1).

### Transfection and plasmid construction

Cells (SW620 and HT-29) were transfected with plasmids or shRNA by using Lipofectamine 2000, according to the manufacturer’s instructions. The sense sequences of PGM1 short hairpin (sh)RNA-shPGM1 were: shRNA1, 5′-GGTCCTGCTCCAGA AG CAATA-3ʹ; shRNA2, 5′-GGGATCATCACTGGTGGTTGG-3ʹ; shRNA3, 5′-GCAGATGGCA GCTGCCAATGG-3ʹ. The sh-negative control (NC)-shCTRL was 5′-CAGTTGAC GAGCAGTGC ATTT-3ʹ. The PGM1 overexpression plasmid (pcDNA4.0-PGM1) and empty plasmid (pcDNA4.0) were obtained from Synbio Technologies Co., Ltd. (Suzhou, China). To construct stable PGM1 knockdown and overexpression cell lines, HT-29 and SW620 cells were treated with lentiviral control-shRNA (shCTRL), lentiviral-PGM1-shRNA-1 (shPGM1), lentiviral- pcDNA4.0-PGM1, lentiviral- pcDNA4.0, and then selected using puromycin (5 μg/mL, Sigma).

### Measurements of cell proliferation and colony formation

The CCK-8 assay (Dojindo Laboratories, Kumamoto, Japan) was used for evaluating cell proliferation. Triplicate samples of cells (2 × 10^3^) were added to 96-well plates and the absorbance of each well at 450 nm was determined each day for three consecutive days. For measurements of colony formation, triplicate samples of treated cells (1.5 × 10^3^) were cultured for 14 days; after which, they were washed twice with PBS, fixed in methanol for 10 min, and stained with 0.1% crystal violet for 10 min.

### EDU proliferation assay

Cell proliferation was measured with an EdU kit (Ribobio, Guangzhou, China). The cells (10 × 10^5^ per well) were inoculated into confocal plates in 50 μM EDU buffer and then incubated at 37 °C for 2 h before fixation (4% formaldehyde, 30 min) and permeabilization (0.1% Triton X-100, 20 min). Next, EdU solution was added, the nuclei were stained with Hoechst staining solution, and the cells were evaluated under a fluorescence microscope.

### Measurement of apoptosis

Transfected cells (HT-29 and SW620 cells, pcDNA4.0 vector, pcDNA4.0-PGM1 vector, NC‑shRNA, or PGM1‑shRNA) were harvested when they reached 90% confluence. The cells were then stained for 10–15 min in the dark at room temperature with 10 μL of Annexin V-APC/7 solution contained in an AAD apoptosis kit (Lianke Biotech co., LTD., Hangzhou, China). Staining results were evaluated by flow cytometry (FACSCalibur, BD Biosciences, Franklin Lakes, NJ, USA).

### TUNEL assay

The cells were washed twice with PBS, fixed in 4% paraformaldehyde and stained; after which, they were visualized using a one-step TUNEL kit (C1089, Beyotime Institute) as previously described [19]. Fluorescence density was analyzed using Image Pro plus 6.0 software. Sections of CRC tumors were dewaxed, incubated with proteinase K (DNase-free, 20 μg/mL; 30 min, 37 °C), washed again, and then incubated with 50 μL of TUNEL reagents (60 min, 37 °C, in the dark). After re-washing in PBS, the sections were evaluated under a confocal fluorescence microscope (Zeiss LSM710, Germany). Apoptotic cells were assessed in 10 randomly selected fields from six sections using Image J software.

### Transwell assays

A 24-well Transwell chamber (8-µm pore size, Corning, NY, USA) was used with or without Matrigel. Transfected cells (5 × 10^4^ in 300 µL of serum-free medium) were inoculated in the upper chamber. Medium containing 10% FBS (700 µL) was added to the lower chamber, and incubated for 24 h at 37 °C. Cells in the lower chamber were fixed with 4% paraformaldehyde and then stained with 0.5% crystal violet. Finally, five fields of each insert were randomly selected for evaluation by light microscopy (magnification, ×200).

### Cell cycle analysis

A cell cycle analysis was performed to determine whether PGM1 was involved in its regulation. Briefly, cells were transfected with pcDNA4.0 vector, pcDNA4.0-PGM1 vector, NC‑shRNA, or PGM1‑shRNA. After harvesting, triplicate samples of cells were fixed (70% ethanol, overnight, 4 °C), stained (PI, 100 μg/mL RNAase), and assessed by flow cytometry.

### Lactate measurements

Cells (2 × 10^5^) were cultured in 12-well plates with a medium change after 10 h. After further incubation for 20 h, lactate was assayed with a Lactate Assay Kit (BioVision, Milpitas, CA, USA). Cellular glycogen levels were measured using a Glycogen Assay Kit (BioVision). Absorbances at 450 nm were read with a microplate reader and lactate concentrations (expressed as mean values ± SD for three independent samples) were calculated from a standard curve.

### ATP measurements

ATP levels were assessed by using an ATP Colorimetric/Fluorometric Assay kit (BioVision) according to the manufacturer’s protocol. The levels of phosphorylated glycerol were quantitated by measuring absorbance at 570 nm.

### Xenograft mice

Athymic BABL/c nude mice (4-weeks old, male) were purchased from the SLAC Laboratory Animal Co. (Shanghai, China). All study protocols were approved by the Institutional Animal Care and Use Committee of Wuhan University Zhongnan Hospital. The mice were housed in a pathogen-free location and randomly assigned to five groups (n = 5 mice per group). SW620 cells (3 × 10^7^, either wild-type or transfected with pcDNA4.0 vector, pcDNA4.0-PGM1 vector, NC‑shRNA, or PGM1‑shRNA) were injected into the left flank. Tumor volumes (calculated from caliper measurements with the formula V = length × width^2^ × 0.5) were determined every third day for 4 weeks. The mice were then sacrificed and the tumors were fixed (4% paraformaldehyde, 24 h) and stained (H&E). Tumor tissues were also immunostained for PGM1 and ki67 using the following antibodies: PGM1 (Abcam; #51248: 1:500) and Ki67 (CST; #9449; 1:400). Images were recorded under a brightfield microscope (Olympus, Tokyo, Japan).

### Statistical analysis

GraphPad Prism 7.0 software (GraphPad, San Diego, CA, USA) was used for data analysis. Data are presented as a mean value ± standard deviation (SD). The t-test was used for assessing differences between two groups and one-way analysis of variance (ANOVA) was used for multiple groups. The chi-square test was used for determining the relationship between PGM1 and CRC clinical features. The cut-off value used for patient division into high and low expression groups was the median PGM1 expression value among all samples. Survival curves were evaluated by the Kaplan–Meier method and differences were measured by the log-rank test. The Cox proportional hazards model was utilized to determinate independent prognostic factors. A P-value < 0.05 was considered to be statistically significant.

## Results

### Identification of PGM1 via TCGA datasets

**Fig. 1:**
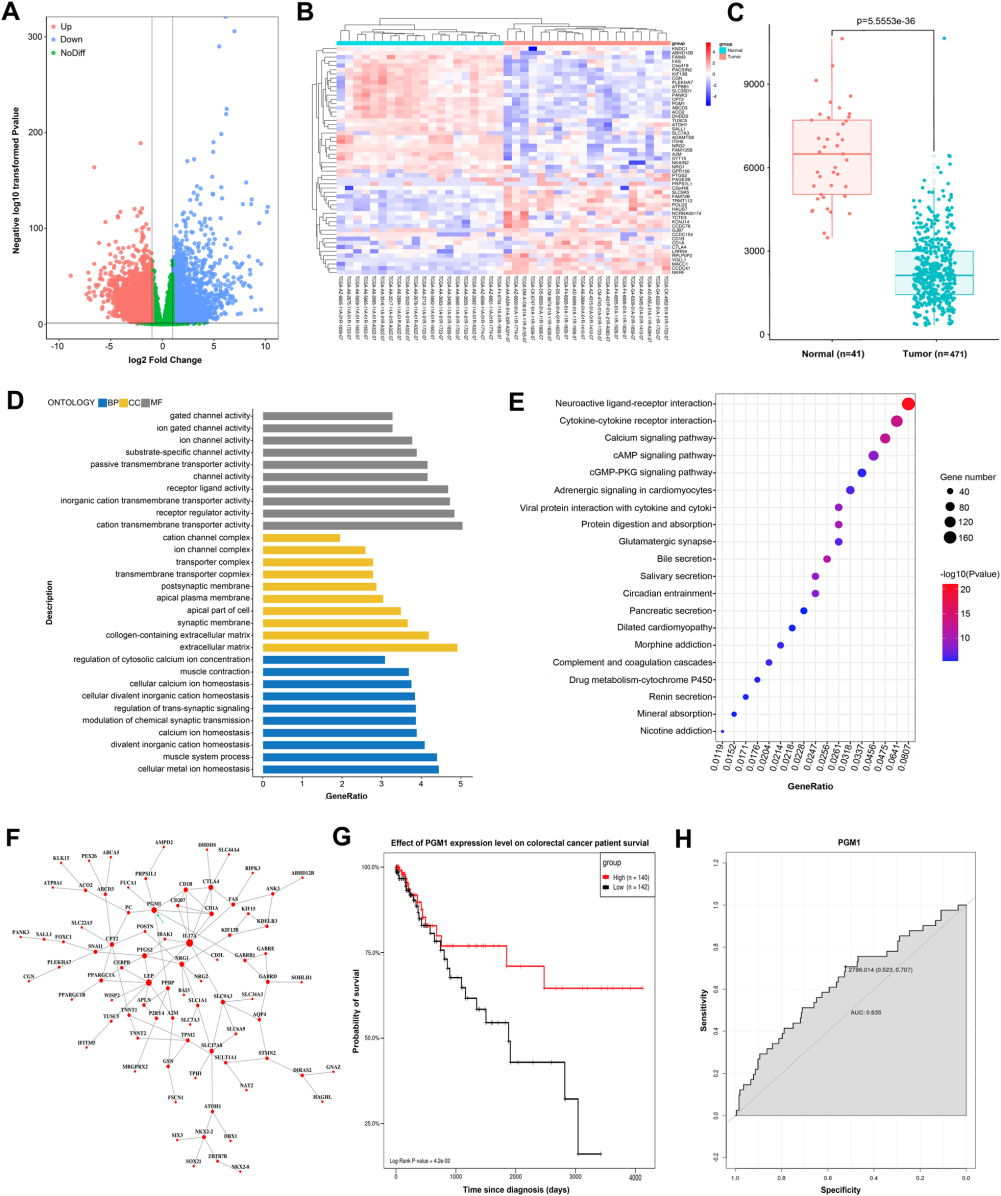
Identification of PGM1 via TCGA datasets. **A** Volcano plot showing the distribution of DEGs in TCGA. Red color shows up-regulated genes in normal tissue while blue color shows down-regulated genes in tumor tissue. **B** A heat map of 54 core genes from the TCGA-COAD dataset comparing CRC tissue and adjoining normal tissue. **C** PGM1 expression in normal control and CRC patients based on samples listed in the TCGA database (matched TCGA normal and GTEx data, P < 0.001). Each dot represents one sample; **D** GO network showing relationships between DEGs and predicted functions. Vertical axis, GO category; horizontal axis, P-values. A lower P-value indicates a greater predicted involvement of the DEG in CRC+. **E** KEGG network showing connections between pathways and DEGs. Vertical axis, pathway classification; horizontal axis, P-value. A lower P-value indicates greater numbers of pathways involving DEGs. **F** PPI (protein–protein interaction) network of DEGs as drawn by Cytoscape. **G** Kaplan–Meier analysis showing the association between PGM1 expression and overall survival among CRC patients. **H** AUC curves with respect to PGM1 gene expression in the TCGA cohort. PGM, phosphoglucomutase; CRC, colorectal cancer; TCGA, The Cancer Genome Atlas; AUC, area under the curve

We initially identified DEGs using the TCGA-CRC data sets. We found that among 5463 DEGs, 2524 (12.7%) were upregulated in normal tissues, and 2939 (14.8%) were downregulated in tumor tissues (Fig. 1A). A heat map (Fig. 1B) shows the top 54 genes that changed the most; in other words, the up- or downregulated genes that were selected for further analysis. GEPIA is an online resource that allows for visualization of TCGA and GTEx data [20]. As shown in Fig. 1C, a high level of PGM1 expression was observed in 41 normal tissues when compared to 471 colorectal tumor tissues (P < 0.0001).

DAVID was used to determine DEG characteristics. A GO analysis showed that DEG biological processes (BP) were significantly enriched in “cellular metal ion homeostasis”, “muscle system process”, and “divalent inorganic cation homeostasis”. DEG cell components (CC) were largely enriched in “extracellular matrix” and “collagen-containing extracellular matrix”, while a molecular function (MF) analysis showed enrichment in “cation transmembrane activity” and “receptor regulator activity” (Fig. 1D). A KEGG pathway determination showed DEG enrichment in “neuroactive ligand-receptor interaction”, “calcium signaling pathway”, and” cytokine-cytokine receptor interaction” (Fig. 1E). Cytoscape was used for drawing a DEG PPI network and identifying significant modules (Fig. 1F).

Furthermore, follow-up data for the enrolled patients were collected for survival analysis. A Kaplan–Meier curve revealed a better overall survival rate among CRC patients with a high-level of PGM1 (Fig. 1G). ROC curves illustrated the diagnostic potential of PGM1 in CRC, with a relatively high sensitivity (AUC = 0.635, Fig. 1H). The aforementioned data suggested that PGM1 could be a prognostic marker for CRC.

### Decreased PGM1expression in CRC was linked to a poor prognosis

**Fig. 2:**
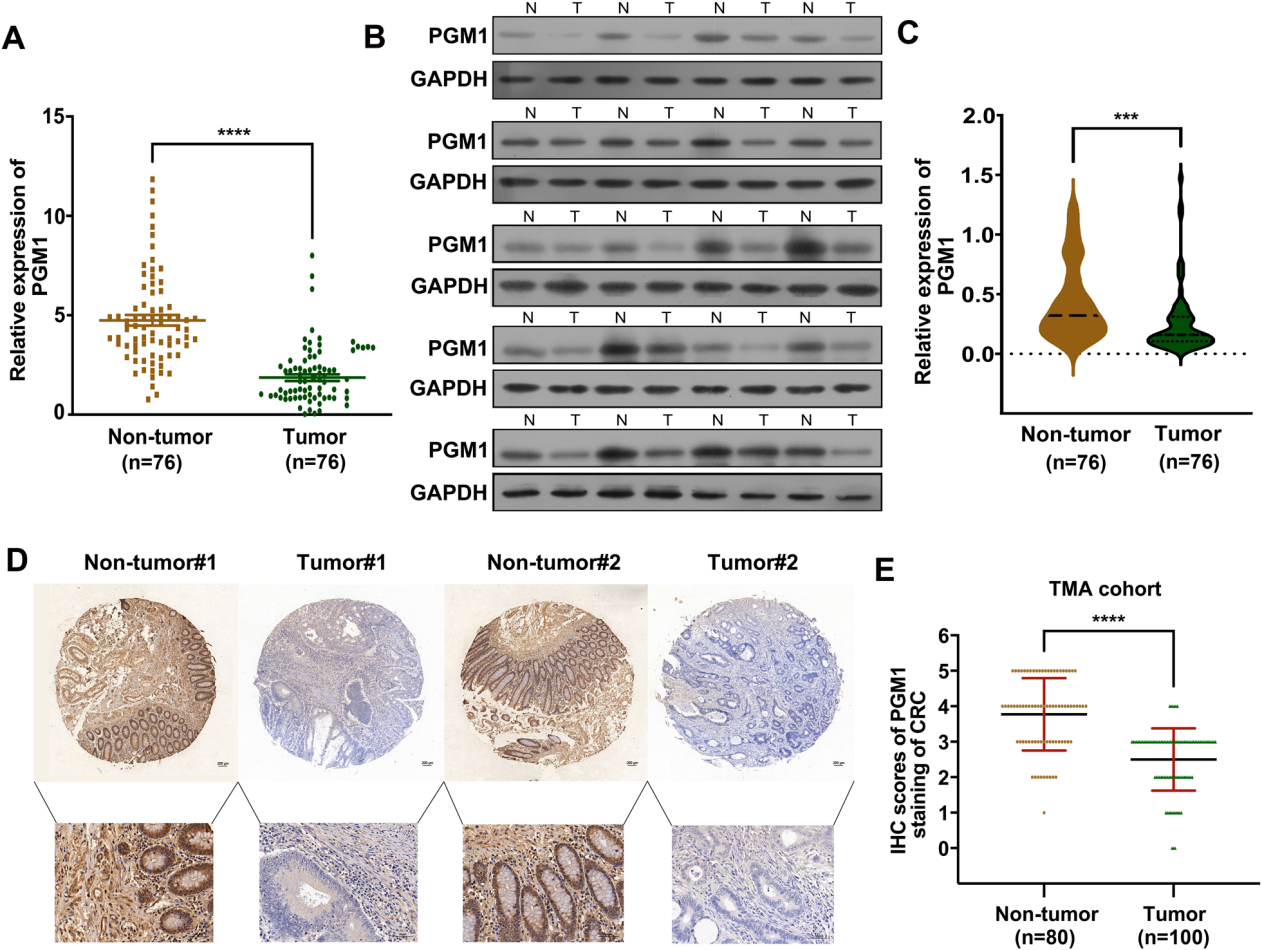
Downregulated PGM1 expression was linked to a poor prognosis. **A** Expression of PGM1 mRNA in CRC and normal tissues as determined using RT-PCR (n = 76). **B** PGM1 protein levels in tumor and non-tumor tissues. **C** PGM1 protein levels in paired tumor and non-tumor samples (n = 76). **D**, **E** PGM1 IHC TMA images contained 100 pairs of tumor and non-tumor tissue (×200, scale bar = 100 μm), respectively. Quantification of cell number is shown. ***< 0.001, ****< 0.0001

To determine the value of the PGM1 level as a CRC biomarker, we used the PCR to measure PGM1 mRNA levels in 76 pairs of tumor and matched adjoining normal tissue. It was observed that PGM1 mRNA levels in the CRC tissues were reduced when compared to the levels in non-CRC tissues (P < 0.001; Fig. 2A); furthermore, this difference was also revealed by WB assays **(**Fig. 2B, C). As verification, IHC staining was used to evaluate PGM1 protein levels in 100 CRC tissues on microarray chips (Fig. 2D), and the integrated optical density (IOD) for PGM1 expression in 100 tumor and normal samples was analyzed using GraphPad Prism software. As shown in Fig. 2E, PGM1 expression was lower in the tumor tissues (P < 0.001). Moreover, the high-PGM1 patient group had a better overall survival rate (P < 0.0001; Additional file 1: Fig. S1) than the low-PGM1 group. These results indicated that PGM1 expression was reduced in CRC tumors and PGM1 expression could affect the survival prognosis of CRC patients.

In order to assess the link between PGM1 levels and clinical characteristics, we collected tumor tissues and demographic data from 76 CRC patients. The median value of PGM1 expression among those tissues was 1.34. On that basis, the 76 samples were divided into high (index > 1.34, n = 38) and low (index ≤ 1.34, n = 38) PGM1 expression groups according to median value. Further analyses were conducted in different subgroups. Table 1 shows a significant difference between low- or high-PGM1 expression and “tumor size” (P = 0.0214), “lymph node metastasis” (P = 0.0027), “clinical stage” (P < 0.0010), and “distant metastasis” (P = 0.0365). However, there was no significant difference between PGM1 expression and “age”, “sex”, or “degree of tumor differentiation”. Furthermore, a multivariate analysis indicated that “lymphatic node metastasis”, “TNM stage”, and “distant metastasis” were independent risk factors for a poor outcome in CRC patients (Table 2). Consistent with results shown in Fig. 1H, a Kaplan–Meier analysis indicated a link between lower PGM1 expression and a poorer overall survival (OS) rate. Taken together, these results indicate that PGM1 expression levels are reduced in CRC patients, and those reductions are linked to a poor prognosis.

**Table 2:** Multivariate analysis of the correlation between PGM1 expression and clinicopathologic parameters

Parameters	EXP (B)	P-value	OR	95% CI
Tumor size (cm): ≥ 5	− 1.114	0.063	0.328	0.101–1.063
Lymphatic node metastasis: positive	− 1.605	0.006	0.201	0.064–0.635
TNM stage: III + IV	− 1.391	0.018	0.249	0.079–0.784
Distant metastasis: yes	− 1.215	0.037	0.297	0.095–0.928

### PGM1-overexpressing and PGM1-knockdown CRC cell lines were successfully constructed

**Fig. 3:**
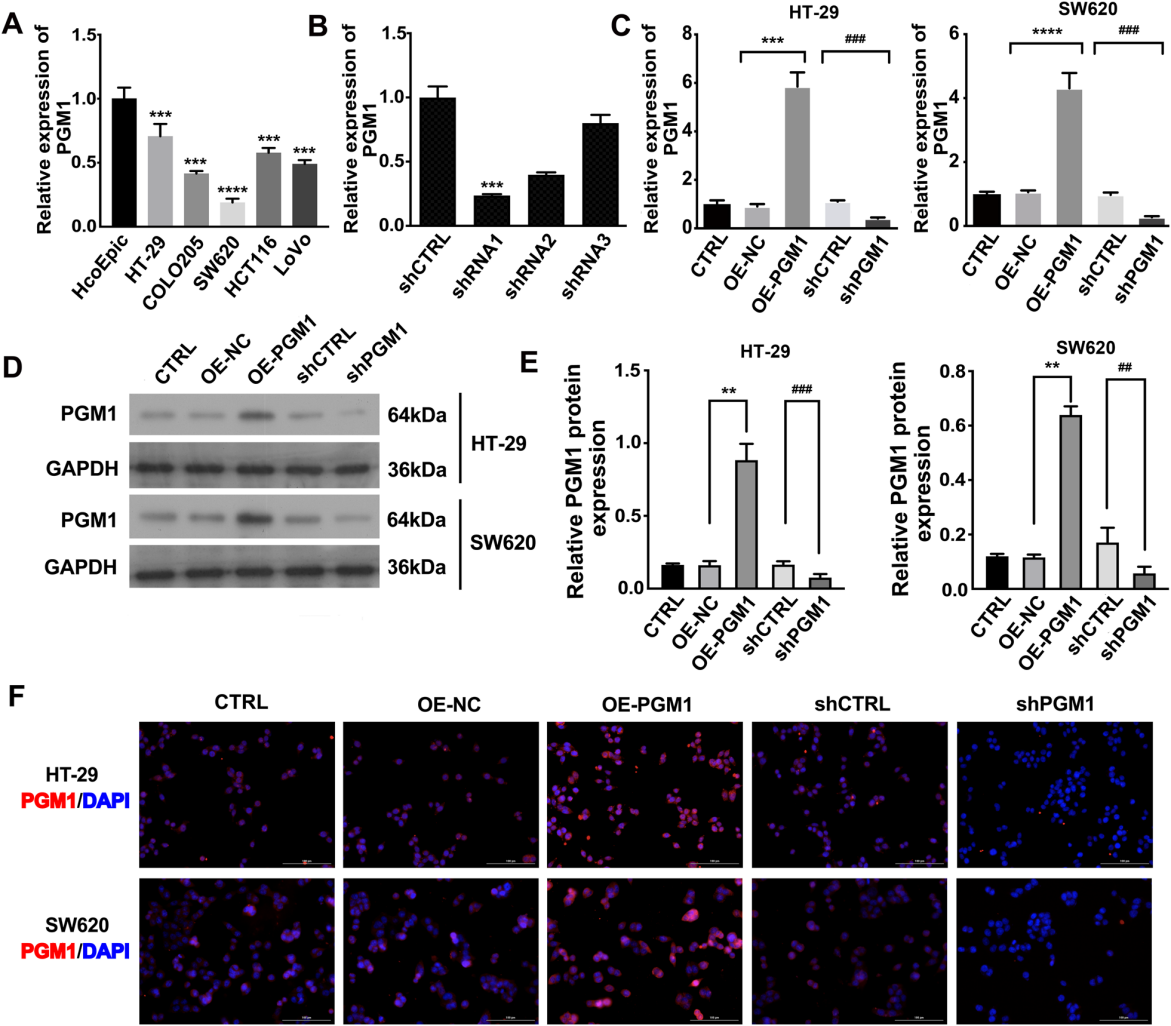
Successful construction of PGM1-overexpression and knockdown cell lines. **A** PGM1 expression in CRC cells as measured by RT-PCR. **B** PGM1 mRNA in control cells and in cells transfected with PGM1 shRNA. ***P < 0.01 vs. shCTRL. **C** PGM1 mRNA expression in cells transfected with pcDNA4.0 vector, pcDNA4.0-PGM1 vector, NC‑shRNA, or PGM1‑shRNA was determined by RT-qPCR. **D**, **E** PGM1 protein expression in the following five groups of transfected cells: CTRL, OE-NC, OE-PGM1, shCTRL, and shPGM1. **F** Images of cells transfected with the aforementioned vectors or shRNAs after being stained for PGM1 expression (red); nuclei are stained blue (DAPI). IOD, integrated optical density; CTRL, negative control; OE, over expression. Data are presented as a mean value ± SD of results obtained from three replicate samples; **P < 0.01, ***P < 0.001, ****P < 0.001; ^##^P < 0.01, ^###^P < 0.001

To further investigate the role of PGM1 and identify the best cell lines for overexpressing or silencing PGM1, PGM1 expression was examined in five CRC cell lines, as well as in HCoEpiC cells. Both RT-PCR and western blotting results showed that PGM1 was most highly expressed in HT-29 cells (P < 0.001), and its lowest expression was in SW620 cells (P < 0.0001; Fig. 3A). Treatment with shRNA-1 was the most effective method of silencing *PGMI* when compared to the negative control group (shCTRL, P < 0.001) (Fig. 3B). Thus, the HT-29 and the SW620 cell lines were selected for silencing PGM1 expression by use of shRNA-1. In addition, we also established CRC cells (SW620 and HT-29) with lentivirus-mediated PGM1 overexpression, which was verified by both qRT-PCR and western blotting (Fig. 3C–E). Moreover, immunofluorescence also confirmed the above experimental results (Fig. 3F). These results all demonstrated a significant suppression of PGM1 mRNA and protein production in sh-PRC1-transfected cells and their over-production in the pcDNA4.0-PGM1 vector transfected cell lines.

### Knockdown of PGM1 promoted the proliferation and arrest of more cells in S phase

**Fig. 4:**
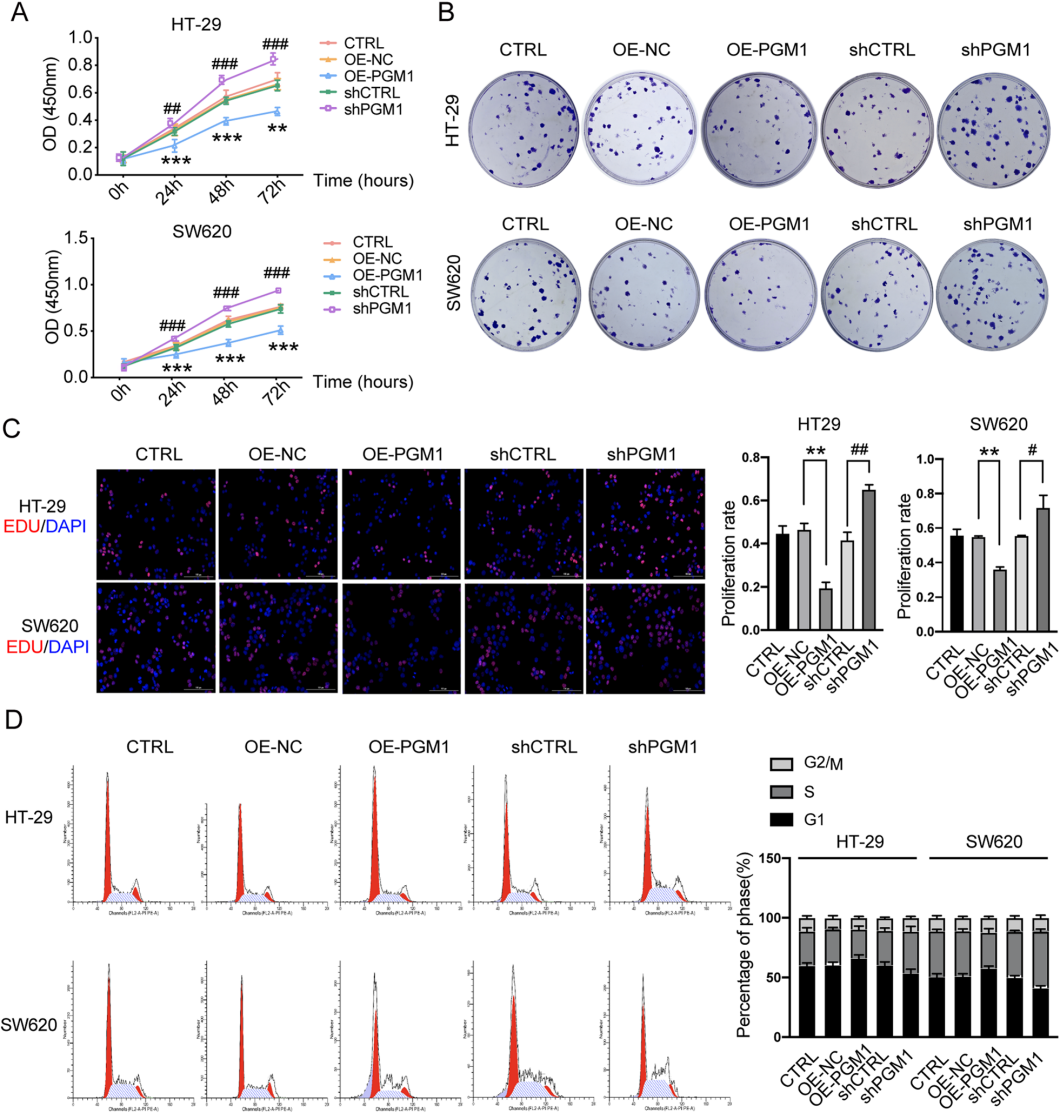
PGM1 knockdown promoted cell proliferation and arrested more cells in S phase. **A** PGM1 overexpression markedly inhibited the proliferation of HT29 and SW620 cells, while knockdown of PGM1 markedly promoted the proliferation of HT29 and SW620 cells. **B** Colony formation abilities of the CTRL, OE-NC, OE-PGM1, shCTRL, and shPGM1 groups of HT-29 and SW620 cells; **C** EdU assays comparing the proliferation of CTRL, OE-NC, OE-PGM1, shCTRL, and shPGM1 groups of HT-29 and SW620 cells (scale bar = 100 μm). **D** Flow chart showing the cell cycle distribution of HT-29 and SW620 cells in the five different groups. Data are presented as a mean value ± SD of results obtained from three replicate samples; *P < 0.05, **P < 0.01, ***P < 0.001; ^##^P < 0.01, ^###^P < 0.001, ^####^P < 0.0001

CCK8, colony-forming, and Edu assays were utilized to investigate the effects of PGM1 on cell proliferation. There was significant inhibition of proliferation in the PGM1 overexpression group when compared to the negative control group (OE-NC) (P < 0.001) (Fig. 4A). Similarly, PGM1-knockdown cells proliferated more rapidly than cells in the shCTRL group (P < 0.001). There were fewer colony numbers among cells transfected with the PGM1 overexpression vector (pcDNA4.0-PGM1) (P < 0.01) (Fig. 4B), while the opposite phenomenon was seen in the shPGM1 group (shPGM1) when compared with the shCTRL group. Moreover, EDU assays also showed reduced cell proliferation in the PGM1 overexpression group, but enhanced proliferation in the PGM1 knockdown group (Fig. 4C).

The cell cycle distribution of cells in the different groups (CTRL, OE-NC, OE-PGM1, shCTRL, and shPGM1) was examined by PI staining. PGM1 silencing resulted in a significant accumulation of S-phase cells and a reduction in G0/G1-phase cells, while the percentages of G2/M-phase cells remained largely unchanged (Fig. 4D). PGM1 overexpression led to reduced percentages of S-phase cells and increased percentages of G1-phase cells. These results suggest that PGM1 suppresses tumor growth by regulating cell proliferation and enhancing the numbers of S-phase cells.

### PGM1 promotes apoptosis in CRC cells

**Fig. 5:**
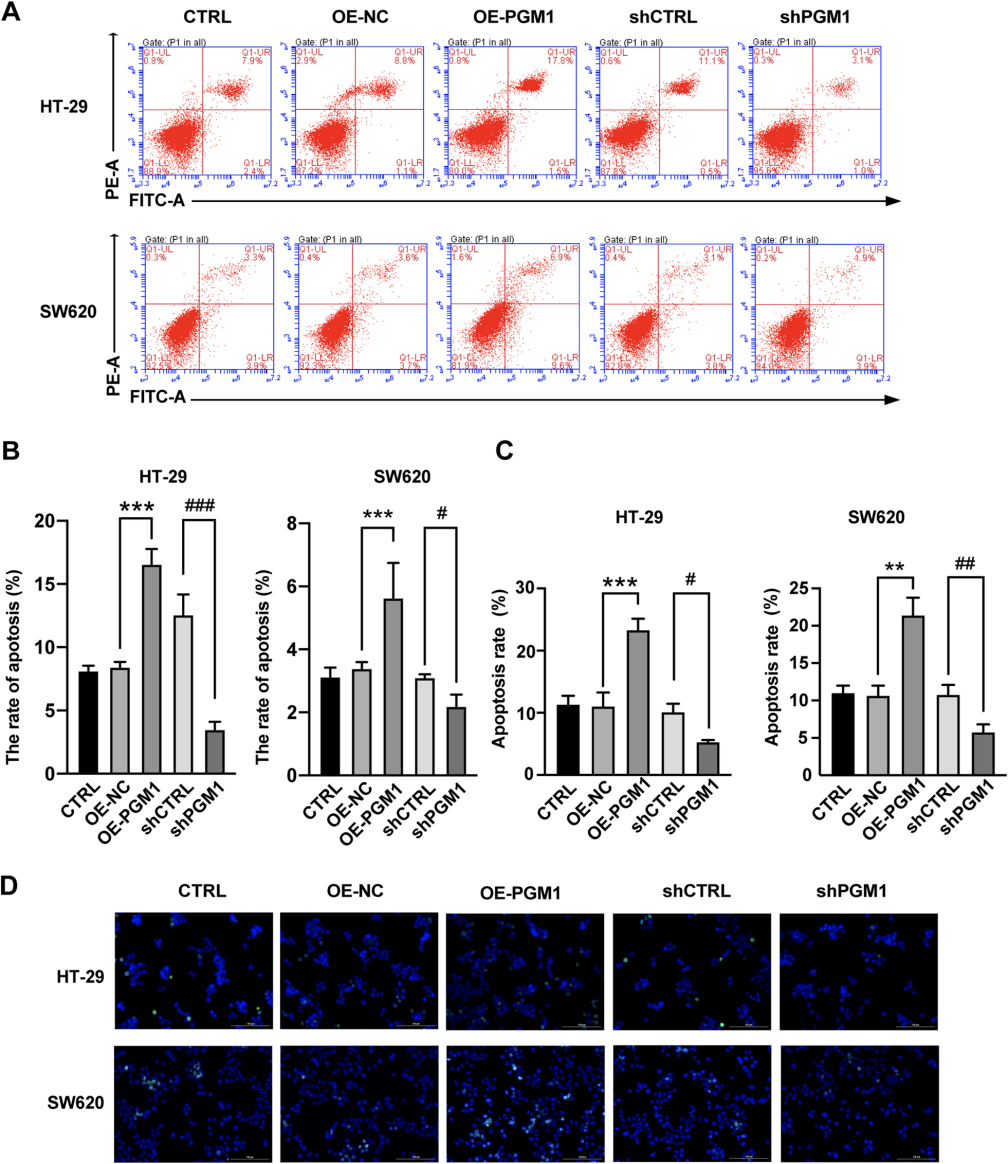
PGM1 promoted apoptosis. **A**, **B** The percentages of apoptotic cells in the different transfection groups (CTRL, OE-NC, OE-PGM1, shCTRL, and shPGM1) were determined by flow cytometry. The percentages of apoptotic cells in the five groups. Bars indicate the mean percentage ± SD. **C**, **D** TUNEL assays for apoptotic HT-29 and SW620 cells in the CTRL, OE-NC, OE-PGM1, shCTRL, and shPGM1 groups (scale bar = 100 μm). HE: hematoxylin–eosin, *P < 0.05, **P < 0.01, ***P < 0.001; ^#^P < 0.05, ^##^P < 0.01, ^###^P < 0.001

Apoptosis is an important factor in CRC pathology. To investigate whether PGM1 acts as a cancer suppressor by modulating apoptosis, we evaluated apoptosis via the FCM and TUNEL assays. Flow cytometry results showed that in contrast to the shCTRL group, there was a reduced rate of apoptosis among the shPGM1 transfected cells, and an increased rate of apoptosis among the PGM1 overexpression cells (Fig. 5A, B). Moreover, TUNEL assays showed that apoptosis was enhanced in the OE-PGM1 group when compared with the OE-NC group (Fig. 5C, D). On the contrary, PGM1 knockdown greatly decreased HT-29 and SW620 cell apoptosis. These data showed that PGM1 promoted the apoptosis of CRC cells in vitro*.*

### PGM1 inhibited cell migration and invasion

**Fig. 6:**
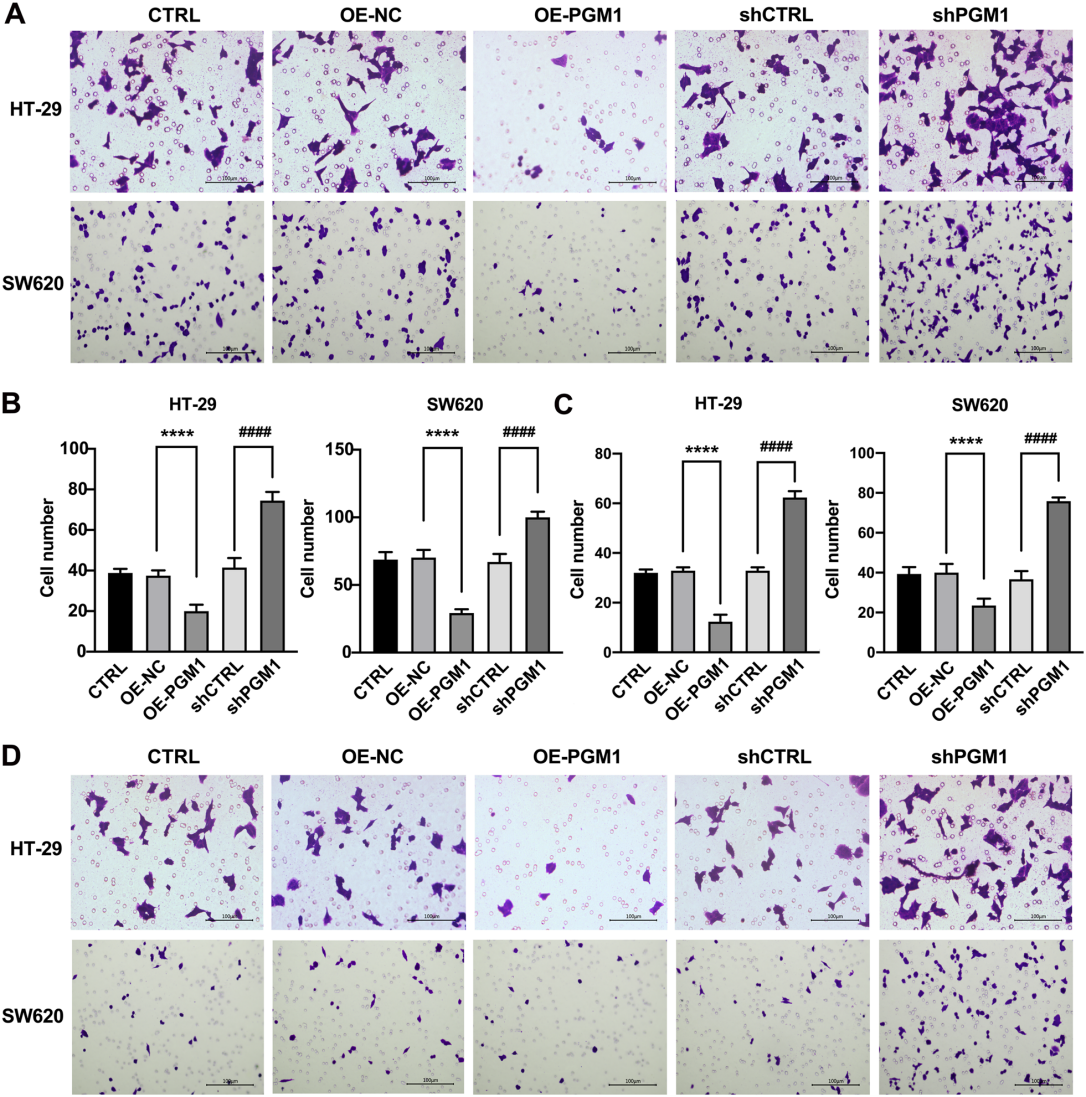
PGM1 inhibited cell invasion and migration. **A** Migration ability of the cell lines. **B** Numbers of migrated cells. **C** Numbers of invaded cells. **D** Invasion ability of the cell lines. ****P < 0.001; ^####^P < 0.0001

Transwell assays were performed to evaluate the invasive and migratory capacities of colorectal cancer cells influenced by PGM1. Consistent with our previous hypothesis, we found that PGM1 up-regulation markedly reduced cell migration (Fig. 6A, B) and invasion (Fig. 6C, D). On the contrary, PGM1 silencing markedly reduced both of those parameters (Fig. 6A–D). These findings further confirmed that PGM1 acts as a tumor-suppressing factor in CRC.

### PGM1 suppressed CRC progression via the PI3K/AKT pathway

**Fig. 7:**
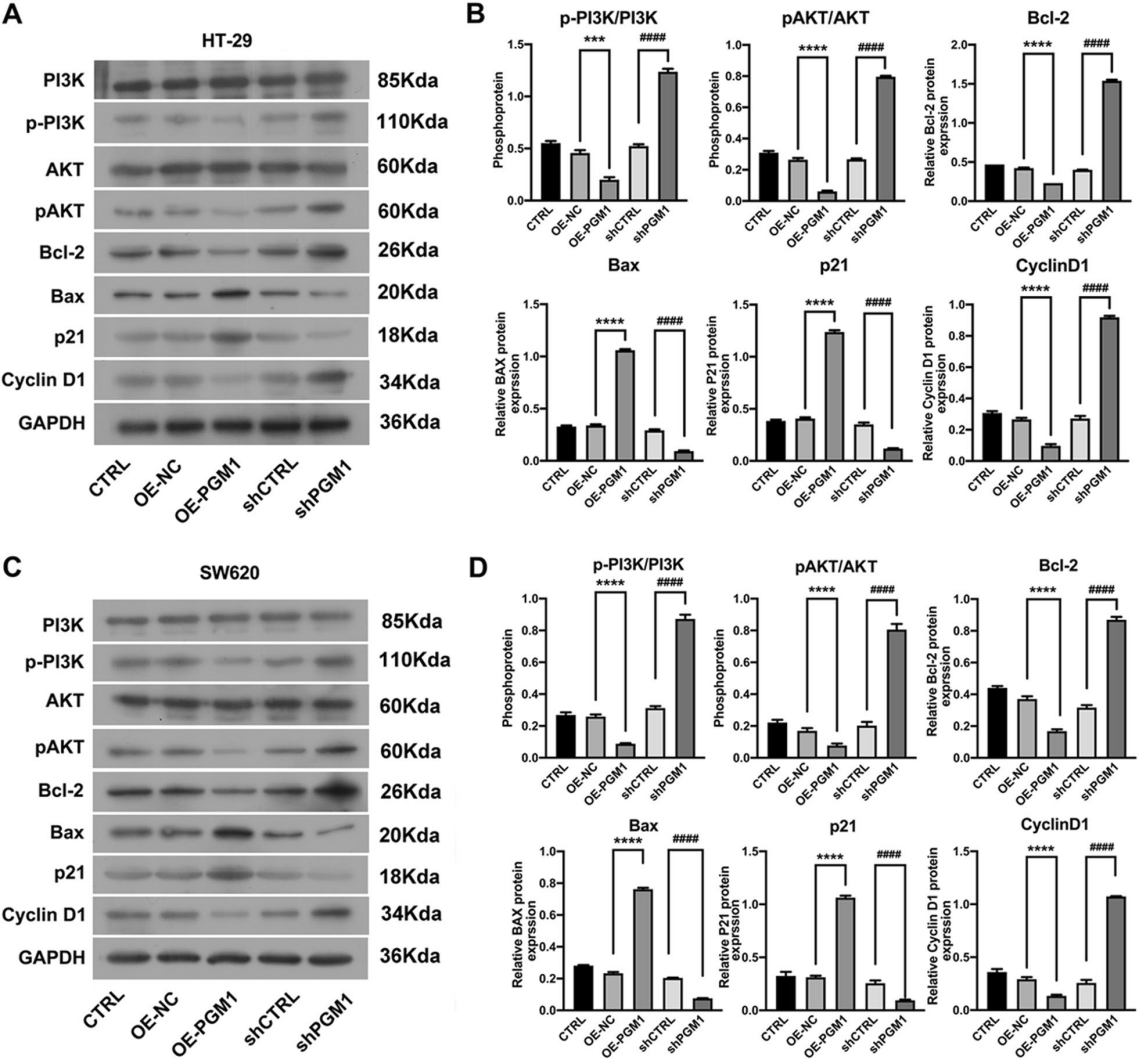
PGM1 action was related to the PI3K/AKT pathway. **A** Levels of PI3K, p-PI3K, AKT, p-AKT, Bcl-2, BAX, P21, and Cyclin D1 protein expression in the five groups of HT-29 cells (CTRL, OE-NC, OE-PGM1, shCTRL, and shPGM1) as determined by western blotting. **B** Protein expression as shown by densitometry. Data were normalized to GAPDH and obtained from three experiments. **C** Levels of PI3K, p-PI3K, AKT, p-AKT, Bcl-2, BAX, P21, and Cyclin D1 protein expression in the five groups of SW620 cells (CTRL, OE-NC, OE-PGM1, shCTRL, and shPGM1) as determined by western blotting. **D** Protein expression as shown by densitometry. Data were normalized to GAPDH and obtained from three separate experiments. ***P < 0.001, ****P < 0.0001; ^####^P < 0.0001

Because we hypothesized that PGM1 might inhibit CRC cell proliferation by downregulating PI3K/AKT signaling, we assessed the levels of p-PI3K and p-AKT in CRC cells. The diminished p-PI3K and p-AKT protein levels induced by PGM1 overexpression substantially increased after the CRC cells were transfected with shPGM1 (Fig. 7A, B). Therefore, we conclude that to some extent, PGM1 inhibits CRC progression by activating the PI3K/AKT pathway.

We also assessed apoptosis-related proteins. We observed that PGM1 positively regulated Bax and negatively regulated Bcl-2 (P < 0.05) (Fig. 7A, B). Thus, it appears that PGM1 exerts a tumor suppressive affect by regulating both apoptosis and the S phase of cells.

In terms of cell cycle-related proteins, we found a down-regulation of G1 inhibitors (p21) (P < 0.0001) and an up-regulation of regulatory proteins (Cyclin D1) in shPGM1-transfected cells (P < 0.0001, Fig. 7A, B). In contrast, p21 levels were increased (P < 0.0001) and Cyclin D1 levels were decreased in response to PGM1 overexpression.

### Knockdown of PGM1 accelerated tumor growth via the PI3K/AKT pathway

**Fig. 8:**
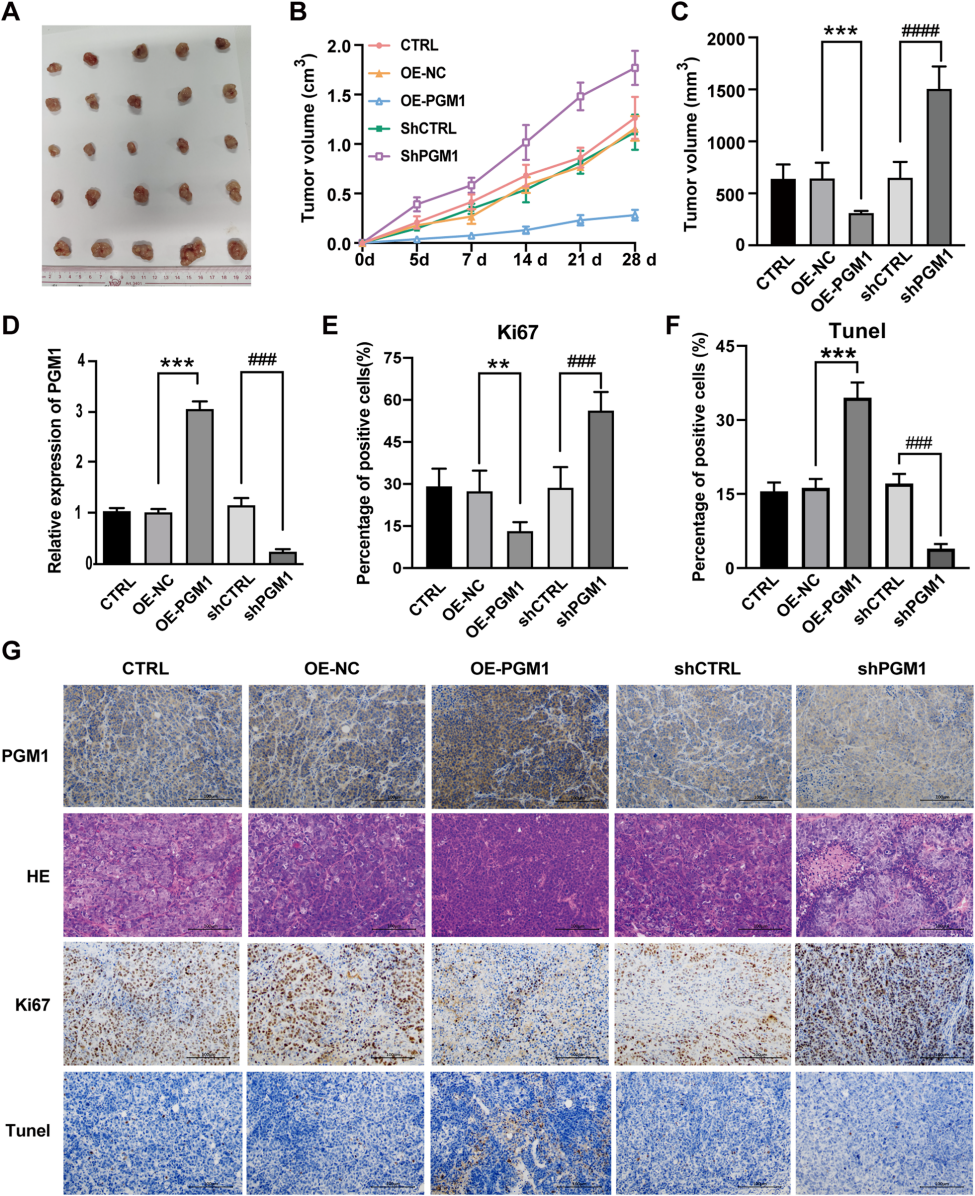
Downregulation of PGM1 accelerated tumor growth in vivo. **A** Tumors formed at 6 weeks post-injection (n = 5/group). Tumors in the CTRL, OE-NC, OE-PGM1, shCTRL and shPGM1 groups were removed upon completion of the study. **B** Tumor growth in the 5 groups was assessed by measurements of tumor volume over time (mean ± SD; n = 25). **P < 0.01. Mice were anesthetized and sacrificed at experimental endpoints. The tumors were subsequently dissected. **C** Tumor volume was monitored in the five aforementioned groups at the endpoints in each experiment. **D** PGM1 mRNA expression in tumors from the CTRL, OE-NC, OE-PGM1, shCTRL, and shPGM1 groups; n = 5. **E** Representative images of PGM1 IHC, H&E, Ki67, and TUNEL staining in the CTRL, OE-NC, OE-PGM1, shCTRL, and shPGM1 groups, respectively. (×200, scale bars = 100 µm). **P < 0.01, ***P < 0.001; ^###^P < 0.001, ^####^P < 0.0001

A mouse tumor model was used to investigate the action of PGM1 in vivo. SW620 cells transfected with pcDNA4.0 vector, pcDNA4.0-PGM1 vector, NC‑shRNA, or PGM1‑shRNA were injected into the mice; after which, PGM1 mRNA expression in five groups of tumors formed by the different treated cells was determined by RT-PCR (Fig. 8D). Consistent with the previous results of cell experiments, it was observed that tumors in the knockdown group (shPGM1) were markedly larger than those in the shCTRL group (P < 0.0001), and tumors in the PGM1 overexpression group (OE-PGM1) were smaller than those in the control group (OE-NC, P < 0.001) (Fig. 8A–C). H&E staining was used to visualize the morphological changes in each group (Fig. 8G). An immunohistochemical analysis of the tumor tissues indicated reduced expression of the differentiation marker Ki67 (P < 0.05, Fig. 8E and G). Moreover, TUNEL assays showed that PGM1 overexpression (OE-PGM1) led to more apoptosis than in the control group (OE-NC), while apoptosis was reduced in the PGM1 knockdown group (shPGM1) when compared with the shCTRL group (Fig. 8F and G). Thus, PGM1 inhibition led to a suppression of CRC growth and enhanced cell differentiation. Furthermore, we used a P13K/AKT inhibitor (LY294002) to block activation of the PI3K/AKT pathway. Cell proliferation increased to its highest level in the shPGM1 group, but this phenomenon was abolished by blocking the PI3K/AKT pathway in the shPGM1 + LY group as detected by CCK8 colony formation assays (Additional file 2: Fig. S2A, B). Western blotting was used to examine changes in p-PI3K and p-AKT expression (Additional file 2: Fig. S2C). Similarly, the increased cell migration and invasion capabilities observed in the shPGM1 group were diminished by LY294002 (Additional file 2: Fig. S2D–G). These results further confirmed that PGM1 inhibits CRC progression by activating the PI3K/AKT pathway.

### PGM1 regulated glycolysis to affect cancer metabolism

**Fig. 9:**
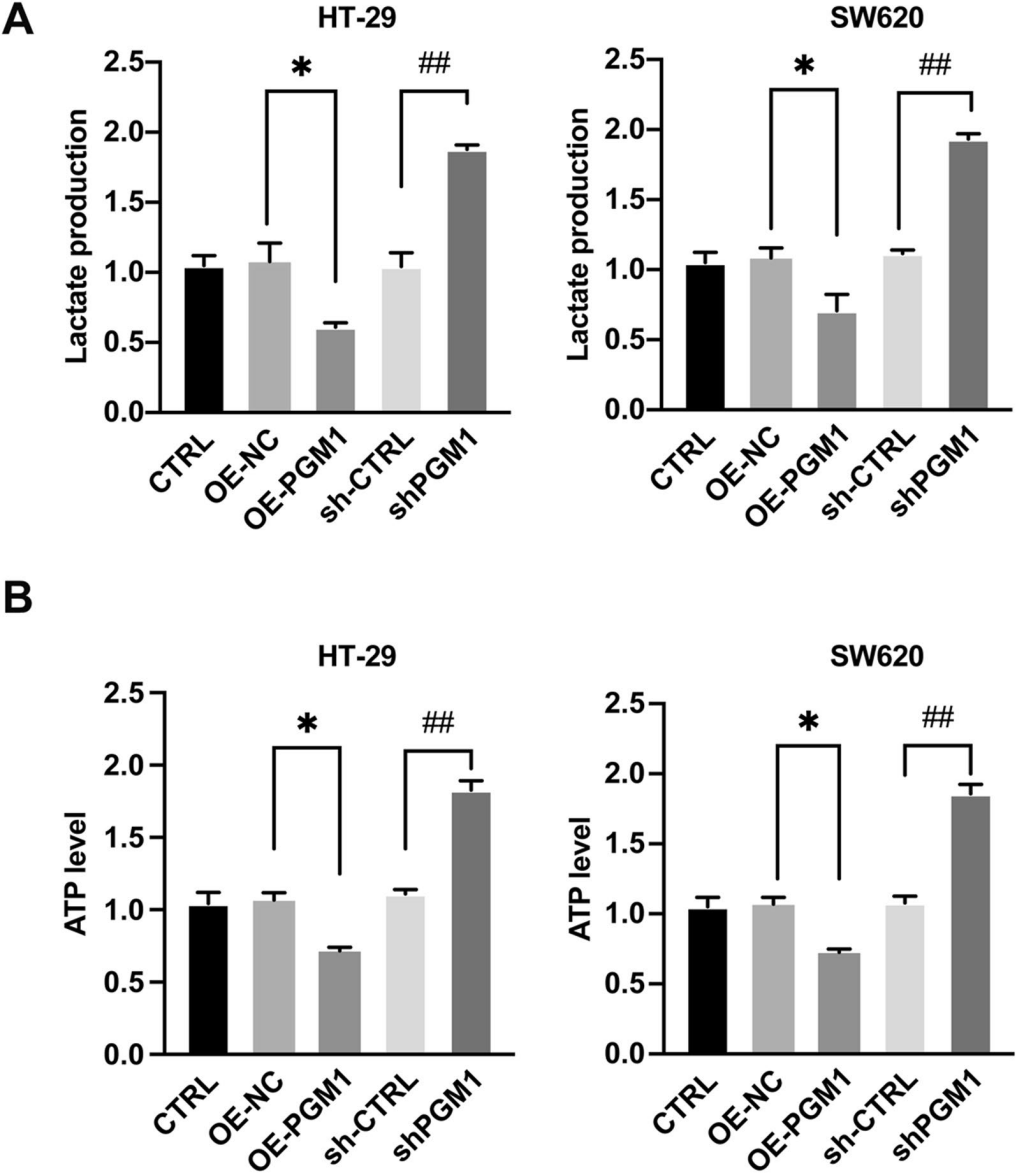
PGM1 inhibited aerobic glycolysis in CRC cells. **A**, **B** Cells were transfected with lentivirus expressing CTRL, OE-NC, OE-PGM1, shCTRL or shPGM1. Culture media was used for determining lactate production (**A**) and ATP levels (**B**) of the cells. Data are presented as a mean value ± SD of results obtained from three separate experiments. *P < 0.05, ^##^P < 0.01

Aberrant metabolism is a major hallmark of cancer. PGM1 may assist in rebalancing glycogen synthesis and glycolysis by reversibly facilitating phosphate transfer during glucose metabolism. Based on that hypothesis, we investigated whether PGM1 played a role in glucose metabolism. It was found that PGM1 overexpression inhibited both lactate and ATP production in HT-29 cells, while PGM1 knockdown produced the opposite effects. Furthermore, these results were also confirmed in SW620 cells (Fig. 9A, B). These results indicated that PGM1 is involved in aerobic glycolysis occurring in CRC cells.

## Discussion

There are five proteins in the PGM superfamily: PGM1, PGM2, PGM2L1, PGM3, and PGM5. Despite their structural similarities, the substrates and functions of these proteins differ [9, 21, 22]. For example, PGM3 is an N-acetylglucosamine triphosphatase that participates in alanine biosynthesis and has anti-neoplastic functions. Targeting of PGM3 inhibits the hexosamine synthetic pathway and has been found to result in growth arrest and apoptosis in breast cancer cells [23]. Furthermore, a blockade of PGM3 expression with sulforaphane was shown to promote apoptosis in prostate cancer cells [24]. PGM5 is highly expressed in muscle connections [25], and its expression level is predictive of overall survival in CRC [10]. The roles of PGM1 in glucose homeostasis and post-translational glycosylation are well characterized, and the enzyme also plays an important role in glucose trafficking by catalyzing the conversion of G1-P to G6-P. PGM1 deficiency is recognized as an inherited metabolic disorder (CDG1T) [21] that is associated with a variety of phenotypes, including exercise intolerance, dilated cardiomyopathy, and liver disease, indicating that PGM1 plays a role in glucose metabolism [26]. However, the role played by PGM1 in cancer has remained poorly understood.

Studies on the role of PGM1 in cancer are limited. Recent investigations have shown that PGM1 blocks liver cancer progression by regulating glucose trafficking [15]. Nevertheless, high levels of PGM1 have been found in lung tumors, and were shown to correlate with a poor prognosis. Li et al. [16] suggested that glucose deprivation results in increased PGM1 expression that enhances cancer progression, suggesting the possibility of targeting PGM1 in treatment of cancer. However, PGM1 does not show consistent expression patterns in different cancer types, and this might be related to the content of muscle.

Cancer metastasis involves factors related to both the tumor and its microenvironment [27]. The microenvironment is comprised of neighboring tumor cells, extracellular matrix, and interstitial tissue [28–30]. The rapid proliferation of tumors requires a significant amount of resources, and tumors are characterized by alterations in their metabolism [31] that involve a reconstruction of energy metabolism [32].

PGM1 is vital for glucose metabolism, and its absence leads to deficiencies in glycogen metabolism, that in turn, result in disease [33–35]. Specifically, *PGM1* encodes for a phosphoglucomutase associated with carbohydrate metabolism [7]. Therefore, we hypothesized that reduced levels of PGM1 might adversely affect energy metabolism, leading to a remodeling of tumor cell physiology with a shift toward the use of aerobic glycolysis. We also demonstrated that a reduction of PGM1 levels in CRC cells stimulates both cellular proliferation and tumor growth by causing the cells to shift from glycogen synthesis and divert glucose to glycolytic pathways. Our results suggest that PGM1 plays a unique, glucose-dependent role in the suppression of CRC tumors. It was recently found that the roles played by PGM1 in glycogen and glucose metabolism are responsible for suppressing the proliferation of cervical and breast cancer cells [36], and our current results support those findings. All these results suggest that PGM1 might promote or inhibit tumor formation based on the type of tumor and its microenvironment.

PI3K/Akt signaling promotes cell proliferation and survival, and is associated with neoplastic transformation and apoptosis inhibition [37]. It is also known that an aberrant expression of proteins in the P13K/Akt pathway is linked to the progression of various cancers [38–40]. Apart from proliferation, the P13K/Akt pathway is also linked to cell migration and autophagy [41, 42]. Therefore, research on PGM1 and whether it targets the PI3K/AKT pathway may be significant for the management of CRC. Here, we demonstrated that the PI3K/AKT pathway is closely linked to PGM1 expression and functions to maintain cancer cell survival and proliferation. This association is important for developing new therapeutic strategies, and indicates that targeting of glycogen metabolism and (or) PGM1 expression may be useful for treating a variety of cancers that show aberrations in these pathways as well as PGM1 expression.

Taken together, these findings show that PGM1 suppresses CRC by regulating glucose translocation via the PI3K/AKT pathway, and suggest PGM1 as a potential target for detecting and treating CRC.

## Conclusion

Our data showed that PGM1 suppresses CRC progression and that PGM1 levels are significantly downregulated in CRC tissues. In addition, lower levels of PGM1 were associated with a poor prognosis. Moreover, overexpression of PGM1 reduced the proliferation, invasion, and migration of CRC cells. Thus PGM1 might be a new biomarker and therapeutic target for CRC.

## Supplementary Information

**Additional file 1: Fig S1.** PGM1 as a prognostic marker. High PGM1 levels were associated with better overall survival (P = 0.0426) in 76 samples collected by our group.

**Additional file 2: Fig S2.** PGM1-mediated tumor suppression was blocked by a PI3K/AKT inhibitor (LY294002) in vitro. **A** CCK8 assay comparing HT-29 and SW620 cell proliferation in the CTRL, shCTRL, shPGM, shCTRL + LY, and shPGM1 + LY groups. **B** Colony formation ability of HT-29 and SW620 cells in the different groups. **C** Levels of PI3K, p-PI3K, AKT, and p-AKT expression in cells in the 5 groups (CTRL, shCTRL, shPGM, shCTRL + LY, and shPGM1 + LYgroups) as determined by western blotting. **D**, **E** Migration ability of cells in the CTRL, shCTRL, shPGM, shCTRL + LY, and shPGM1 + LY groups as detected by Transwell assays. **F**, **G** Invasion ability of cells in the CTRL, shCTRL, shPGM1, shCTRL + LY, and shPGM1 + LY groups as detected by Transwell assays. **P < 0.01, ***P < 0.001; ##P < 0.01, ###P < 0.001. &&&P < 0.01, &&&P < 0.001.

## Data Availability

The datasets used in this study are available from the corresponding author upon reasonable request.

## Abbreviations

PGM: Phosphoglucomutase
CRC: Colorectal cancer
TCGA: The Cancer Genome Atlas
DEG: Differentially expressed genes
TMA: Tissue microarray
