# Supplementary figures and images for: PGM1 suppresses colorectal cancer cell migration and invasion by regulating the PI3K/AKT pathway
Source: Cancer Cell Int. 2022 May 25;22:201. doi: 10.1186/s12935-022-02545-7 (PMC9134613; doi:10.1186/s12935-022-02545-7)

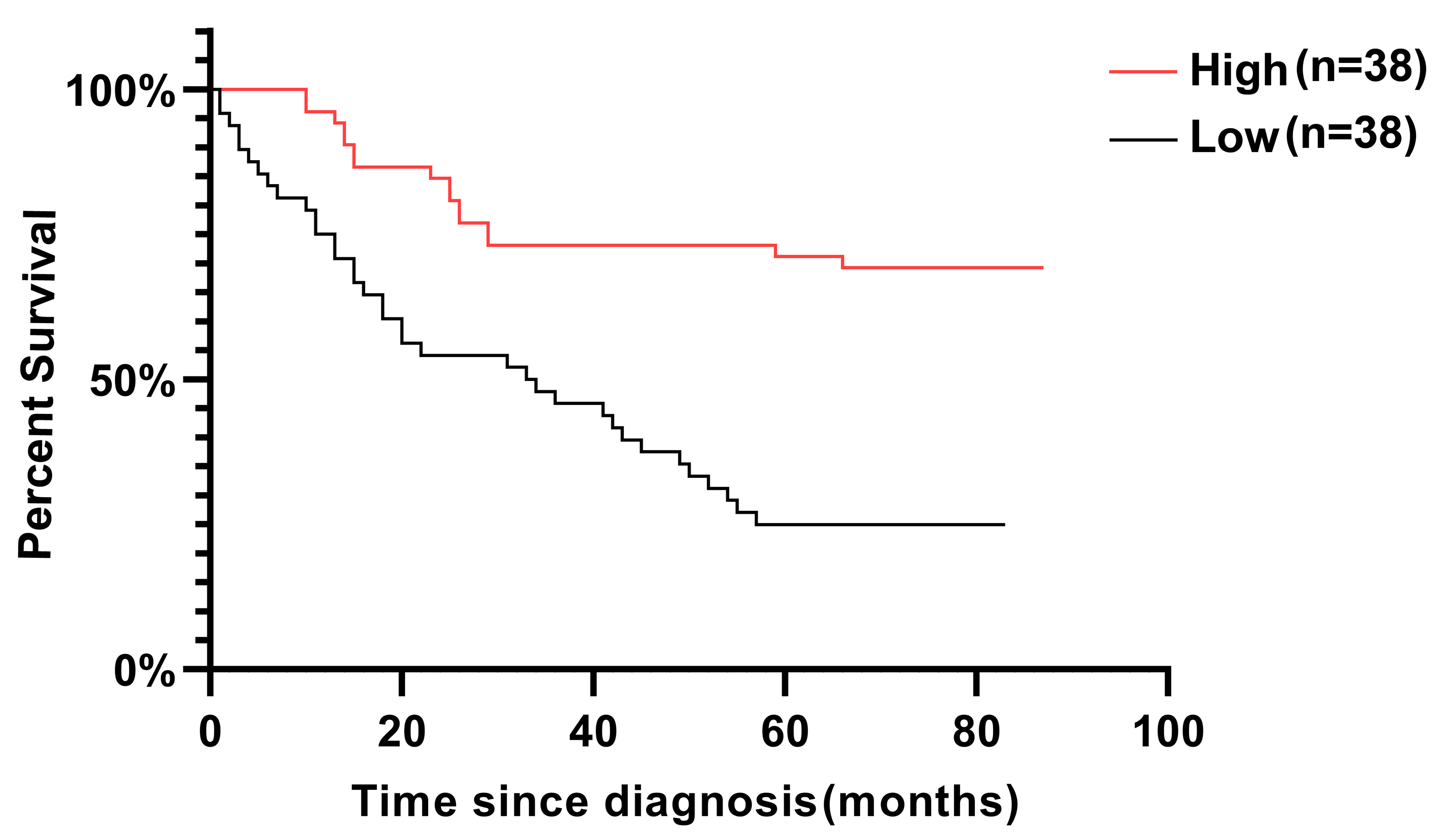

Supplement: Supplementary file 1 — Additional file 1: Fig S1. PGM1 as a prognostic marker. High PGM1 levels were associated with better overall survival (P = 0.0426) in 76 samples collected by our group. [file 12935_2022_2545_MOESM1_ESM.tif]

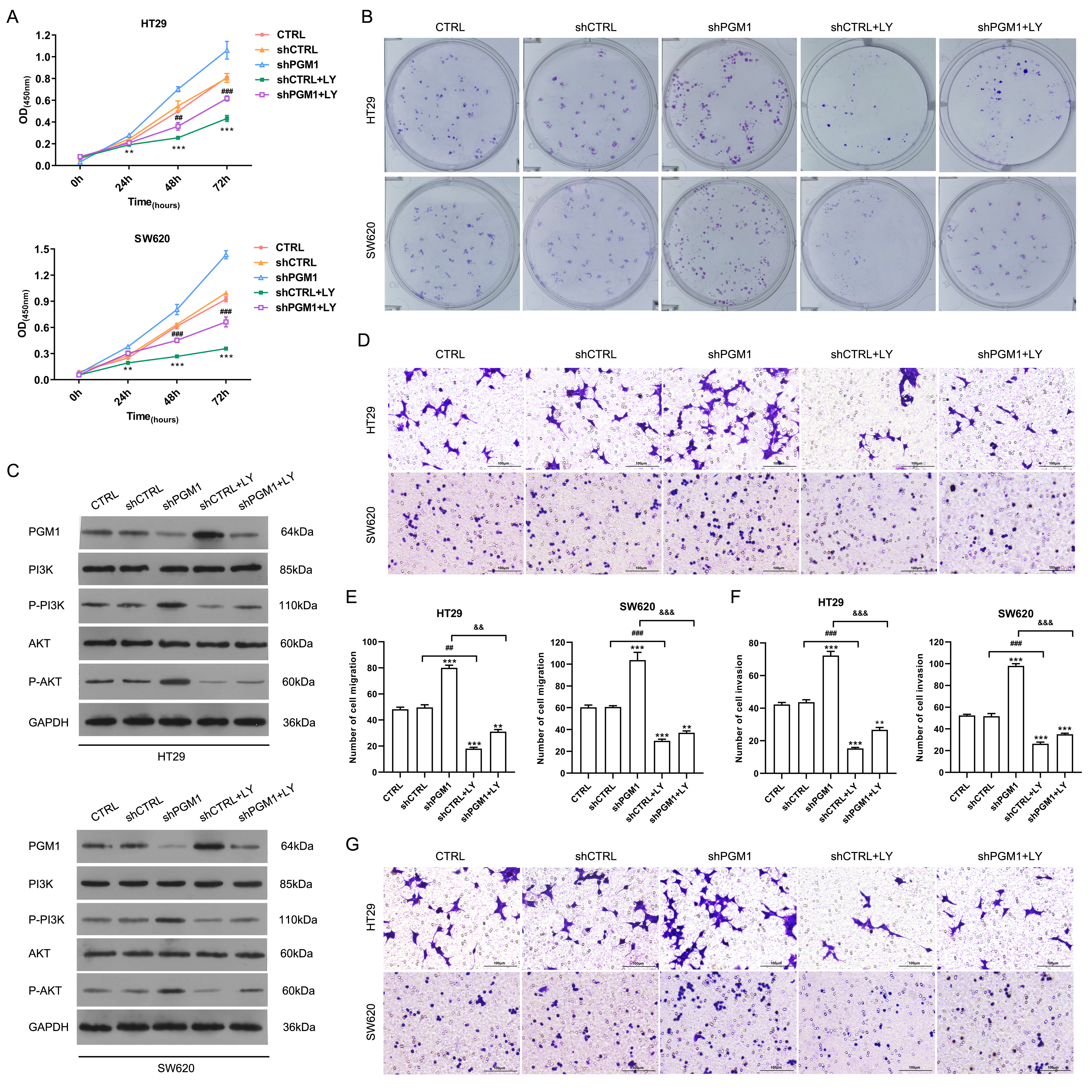

Supplement: Supplementary file 2 — Additional file 2: Fig S2. PGM1-mediated tumor suppression was blocked by a PI3K/AKT inhibitor (LY294002) in vitro. A CCK8 assay comparing HT-29 and SW620 cell proliferation in the CTRL, shCTRL, shPGM, shCTRL + LY, and shPGM1 + LY groups. B Colony formation ability of HT-29 and SW620 cells in the different groups. C Levels of PI3K, p-PI3K, AKT, and p-AKT expression in cells in the 5 groups (CTRL, shCTRL, shPGM, shCTRL + LY, and shPGM1 + LYgroups) as determined by western blotting. D, E Migration ability of cells in the CTRL, shCTRL, shPGM, shCTRL + LY, and shPGM1 + LY groups as detected by Transwell assays. F, G Invasion ability of cells in the CTRL, shCTRL, shPGM1, shCTRL + LY, and shPGM1 + LY groups as detected by Transwell assays. **P < 0.01, ***P < 0.001; ##P < 0.01, ###P < 0.001. &&&P < 0.01, &&&P < 0.001. [file 12935_2022_2545_MOESM2_ESM.tif]
